# Imidazo[1,5-*a*]pyridine-Based Fluorescent Probes: A Photophysical Investigation in Liposome Models

**DOI:** 10.3390/molecules27123856

**Published:** 2022-06-16

**Authors:** Giacomo Renno, Francesca Cardano, Giorgio Volpi, Claudia Barolo, Guido Viscardi, Andrea Fin

**Affiliations:** 1 Department of Chemistry, University of Torino, Via P. Giuria 7, 10125 Torino, Italy; giacomo.renno@unito.it (G.R.); francesca.cardano@unimi.it (F.C.); claudia.barolo@unito.it (C.B.); guido.viscardi@unito.it (G.V.); 2 NIS Interdepartmental Centre and INSTM Reference Centre, University of Torino, Via G. Quarello 15/a, 10125 Torino, Italy; 3 Department of Drug Science and Technology, University of Torino, Via P. Giuria 9, 10125 Torino, Italy

**Keywords:** imidazo[1,5-*a*]pyridine, fluorescence, large Stokes shift, liposome, membrane probes

## Abstract

Imidazo[1,5-*a*]pyridine is a stable scaffold, widely used for the development of emissive compounds in many application fields (e.g., optoelectronics, coordination chemistry, sensors, chemical biology). Their compact shape along with remarkable photophysical properties make them suitable candidates as cell membrane probes. The study of the membrane dynamics, hydration, and fluidity is of importance to monitor the cellular health and to explore crucial biochemical pathways. In this context, five imidazo[1,5-*a*]pyridine-based fluorophores were synthesized according to a one-pot cyclization between an aromatic ketone and benzaldehyde in the presence of ammonium acetate and acetic acid. The photophysical features of prepared compounds were investigated in several organic solvents and probes **2**–**4** exhibited the greatest solvatochromic behavior, resulting in a higher suitability as membrane probes. Their interaction with liposomes as artificial membrane model was tested showing a successful intercalation of the probes in the lipid bilayer. Kinetic experiments were carried out and the lipidic phase influence on the photophysical features was evaluated through temperature-dependent experiments. The results herein reported encourage further investigations on the use of imidazo[1,5-*a*]pyridine scaffold as fluorescent membrane probes.

## 1. Introduction

Imidazo[1,5-*a*]pyridine is a well-known and widely tested scaffold for the development of innovative compounds in several fields. It gained an increased attention during the last decades [1,2] as versatile moiety able to fulfill different requirements such as a compact shape, emissive properties, and photo/thermal stability among others [3]. The synthesis is straightforward and can be achieved by different approaches in an easily up-scalable manner [4,5,6,7]. Imidazo[1,5-*a*]pyridine compounds can be used as (i) candidates in medicinal chemistry [8,9]; (ii) ligands for a plethora of coordination complexes [10,11]; (iii) chemical sensors [12,13]; (iv) smart materials in optoelectronics [3,14]; and (v) fluorophores for bioimaging [15,16,17]. In particular, the study of the membrane dynamics in living cells led to the development of many fluorescent probes based on different action strategies (e.g., solvatochromic compounds, flippers) [18,19,20]. The ability to discriminate between several lipid phases and to visually highlight the fluidity and hydration of the membrane is of importance to shed light on the cell health as well as on many crucial biochemical pathways. Historically, Weber and Lakowicz have pioneered the design of small probes suitable for the investigation of biological domains such as proteins and membranes by functionalizing a naphthalene core to introduce unique photophysical features in the so called Prodan, Laurdan, and Patman dyes [21,22]. Nowadays many fluorescent probes, specifically designed for the investigation of lipid bilayer membrane, are available on the market and are extensively used by the scientific community. Lipophilic probes, not covalently linked to membrane building blocks, such as DPH, ANS, and DCVJ, have been used to investigate membrane fluidity [23,24,25] due to their fluorogenic features. More polar fluorophores such as NBD, Rho-101, and DPE have been reported to label or replace the polar head groups of phospholipids to explore the outer membrane surface [26,27,28]. Well-known fluorophores such as pyrene and fluorene have been covalently linked on-chain and in chain-end to investigate the interior of the membrane [29,30]. More recently, bright dyes absorbing and emitting in the near infrared region of the spectra have been proposed to shed light on membrane dynamics [31]. Finally polarizable and planarizable oligo-aromatic probes have been developed to visualize membrane hydration and mechanical compression in living cells [32]. The design and investigation of molecules, suitable for application in bioimaging is a dynamic field in which novel probes are constantly prepared and evaluated to provide novel tools for basic and applied science. The present manuscript introduces new fluorophores based on imidazo[1,5-*a*]pyridine as potential probes for application in the bilayer imaging. To the best of our knowledge, any imidazo[1,5-*a*]pyridine had ever been tested in biological applications so far. The straightforward synthesis, the easily functionalization of the core with various groups (e.g electron donating or withdrawing moieties, hydrophilic or lipophilic groups) suggests broad range of applicability of this family of probes in the domain of chemical biology. Therefore, the present work aims to provide a preliminary evaluation of the biocompatibility and potential as membrane fluorophores of a first generation of imidazo[1,5-*a*]pyridine probes rather than proposing novel dyes with feature comparable with commercially available probes.

**Figure 1:**
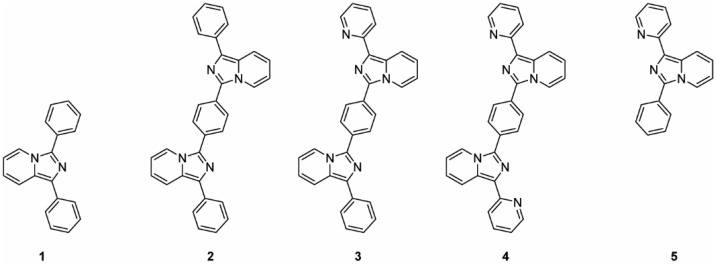
Structures of the synthesized compounds (**1**–**5**).

In this context, five emissive probes were prepared from the imidazo[1,5-*a*]pyridine scaffold: two monomeric units, **1** and **5**, and three dimeric products **2**–**4**, as reported in Figure 1. The synthesis of the asymmetric bis-imidazo[1,5-*a*]pyridine **3** is herein reported for the first time along with the photophysical analyses in comparison with the corresponding symmetric analogs **2** and **4**. All of the prepared compounds were optically evaluated and the dimeric species were tested as fluorescent membrane probes using two different liposomal model systems as cell membrane mimics.

## 2. Results

### 2.1. Synthesis

**Figure 2:**
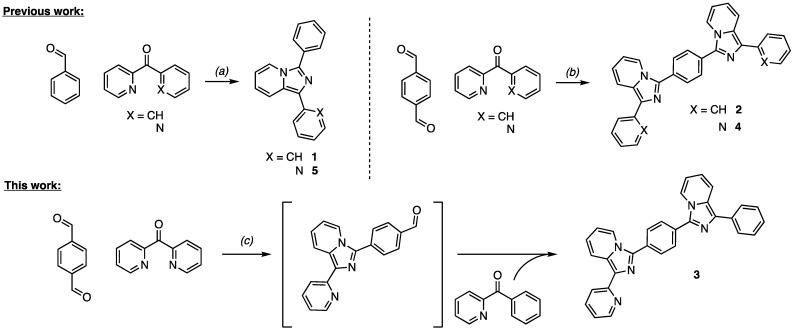
Synthetic approach toward the prepared compounds. (**a**) ammonium acetate, acetic acid, 110 °C, 5 h (**1**: 78%, **5**: 80%); (**b**) ammonium acetate, acetic acid, 118 °C, 12 h (**2**: 96%, **4**: 96%); (**c**) 1. ammonium acetate, acetic acid, 118 °C, 6 h; 2. (phenyl(pyridin-2-yl)methanone, 118 °C, 12 h (78%).

Products **1**, **2**, **4**, and **5** were synthesized by a direct cyclization of phenyl(pyridin-2-yl)methanone (**1** and **2**) or 2,2′-dipyridylketone (**4** and **5**) with different aromatic aldehydes in the presence of ammonium acetate [5,33,34,35]. The heterocyclicazion was pursued in acidic conditions using the ammonium acetate as nitrogen source for the final ring closure as previously reported [5]. Varying the aromatic aldehydes in refluxing acetic acids provides easy access to a large number of both imidazo[1,5-*a*]pyridine and bis-imidazo[1,5-*a*]pyridine. Benzaldehyde reaction with diaryl ketones gives access to the mono-imidazo[1,5-*a*]pyridine products **1** and **5**. The replacement of the benzaldehyde with a bifunctional analogs as the terephthalaldehyde allows the preparation of the symmetic, **2** and **4**, and the asymmetric **3** bis-imidazo[1,5-*a*]pyridine derivatives. The symmetrical bis-imidazo[1,5-*a*]pyridines **2** and **4** were obtained by reacting either the phenyl(pyridin-2-yl)methanone or the 2,2′-dipyridylketone with terephthalaldehyde in stoichiometric ratio 2:1 (Figure 2). To the best of our knowledge only the preparation of symmetrical bis-imidazo[1,5-*a*]pyridine compounds have been reported, following the above mentioned strategy or by varying the acidic environment (e.g., succinic, maleic, phthalic or trimesic acids) [36] or the dialdehydes (e.g., terephthalaldehyde and isophthalaldehyde) [37]. The so obtained products could be considered alternative to polypyridine, polypyrrole, and polyimidazole behaving as promising multidentate scaffolds or ligands for organometallic and supramolecular design.

Herein, for the first time, the synthesis of the asymmetric bis-imidazo[1,5-*a*]pyridine product **3** is reported, by coupling the terephthalaldehyde with two different aryl ketones in a stoichiometric ratio 1:1:1. The present single step coupling of three components allows the introduction of different chemical groups to modulate the π-system features and the functionalization with electron rich or electron poor moieties by straightforward employment of di-aldehydes and substituted aryl ketones. More in detail, the asymmetric scaffold **3** was obtained in a straightforward manner by promoting a first reaction over 6 h in acetic media between the di(2-pyridyl)ketone and terephthalaldehyde followed by the addition of the phenyl(pyridin-2-yl)methanone, providing after 12 h the final product as a yellow powder.

### 2.2. Optical Features

**Figure 3:**
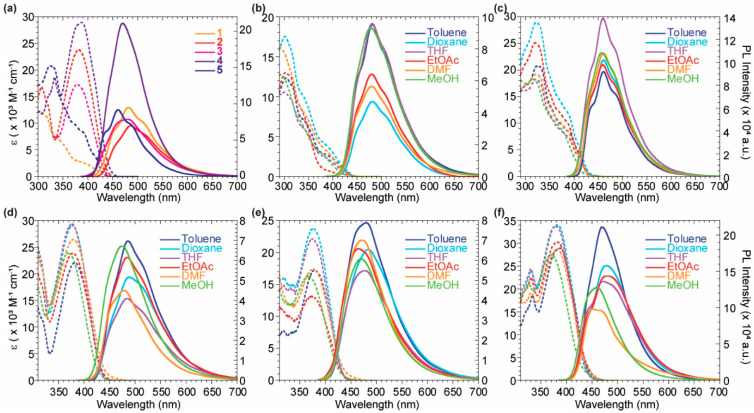
Photophysical properties of the reported probes **1**–**5**. (**a**) Absorption (dashed) and emission (solid) spectra of compounds **1**–**5** in toluene. (**b**–**f**) Absorption (dashed) and emission (solid) spectra in different solvents for compounds **1** (**b**), **5** (**c**), **2** (**d**), **3** (**e**), **4** (**f**). The emission spectra were normalized to 0.1 intensity at the excitation wavelengths.

The absorption and emission spectra of the whole series of probes were acquired in toluene and depicted in Figure 3a. While the emission spectra of all the compounds are centered around 480 nm, the absorption maximum results batochromically shifted when moving from the monomeric species **1** and **5** to the dimeric ones **2**, **3**, and **4** due to an increased conjugation. The formers’ absorption band is centered around 305–325 nm, while the latters’ one moves to 380 nm, gaining a more significant contribution in the visible range. As noticed by Volpi et al. [36,37], the substitution of the benzene ring of **2** with a pyridinil group in **4** increases the quantum yield along with a bare blue-shift of the emission spectra. The preparation of the asymmetrical compound **3** confirmed the above considerations and highlights the possibility to tune the quantum yield value by modulating the number of the pyridine rings introduced in the scaffold. The solvatochromic features of all the reported probes were evaluated (Figure 3b–e) and are listed in Table 1. The highest fluorescence quantum yields (QY) for the dimeric compounds **2**–**4** were observed in apolar environments, such as toluene. The symmetric dipyridil analog **4** was characterized by a good QY of 0.38, which was three times more emissive than the dyphenyl analog **2** showing a QY of 0.12. The asymmetrical scaffold **3** shows an intermediate behaviour, with a QY of 0.18, in agreement with the above hypoteses. All the probes exhibited a wide Stokes shift, higher than 5000 cm^−1^. In general, imidazo[1,5-*a*]pyridine derivatives are well known in the literature for a wide Stokes shift useful for different application such as down-shifting conversion [33,38].

The monomeric species **1** and **5** showed negligible solvent effects on their optical features, due to the low charge-transfer character of the ground state [36]. The probes **2**, **3** and **4** highlighted a higher solvatochromic behaviour, as evidenced by the linear fit of the emission maximum wavenumbers against the E_T30_ polarity scale (Figure S1 in Supplementary Materials). Moreover, in a polar protic solvent as methanol, a clear hypsochromic shift of the emission maxima was present for all the bis-imidazo[1,5-*a*]pyridine derivatives. This effect was more remarkable in presence of one or two pyridine rings in **3** and **4**, most likely due to hydrogen bond interaction of the solvent with the aromatic pyridil nitrogen. These considerations highlight again the notable tunability of the optical features by simple modifications of the number of pyridine rings in the system.

Previously reported data on the imidazo[1,5-*a*]pyridines’ lifetime shows uniformity with values spanning from 2 to 8 ns [17,35]. Compounds **1** and **5** have been previously investigated, revealing lifetime values of 5.8 and 4.6 nm, respectively. Similarly, eight multiple imidazo[1,5-*a*]pyridine derivatives have been previously reported showing similar values in a range of 2–7 ns, thus demonstrating a stringent uniformity in the lifetime data [36]. To the best of our knowledge, imidazo[1,5-*a*]pyridines’ lifetime shows good uniformity due to the similar nature of the excited state as widely previously reported for these heterocyclic scaffolds. Additionally, the possible aggregation in water was evaluated for the dimeric compounds **2**–**4**, aiming to investigate the effects on the photophysical features. In this context, several measurements were carried out in THF with different amount of water (Figure S2). The absorption spectra show a hypochromic effect when increasing the amount of water, along with a peak broadening that becomes more significant in pure water and with the 20% of THF. All the probes still exhibit a clear absorption peak in pure water, suggesting the presence of stable aggregates, while the emission of **2** and **3** is almost completely quenched. Moreover, the shape and position of the emission peaks are characterized by a bathochromic trend while increasing the amount of THF in water for all the investigated probes. This phenomenon highlights the fluorogenic character of these compounds, making them suitable candidate for applications as probes in biological systems. The fluorogenic behavior of a membrane probe can ensure low background noise with beneficial effects on the signal/noise ratio.

### 2.3. Liposomes

**Figure 4:**
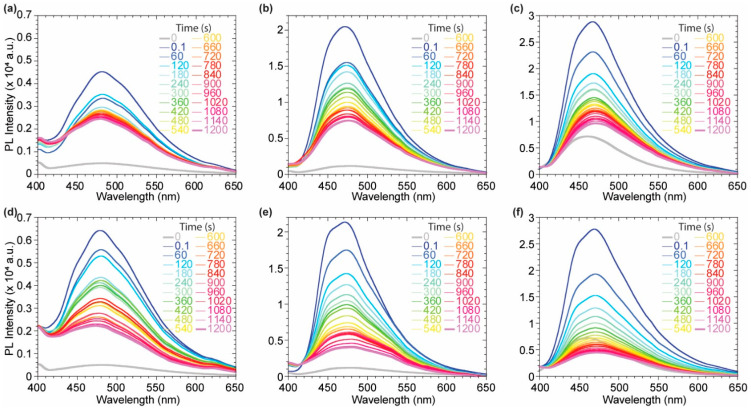
Kinetic experiments in DOPC (**a**–**c**) and DPPC (**d**–**f**) for compounds **2** (**a**,**d**), **3** (**b**,**e**), **4** (**c**,**f**). Probes concentration during the measurement: 7.28 × 10^−7^ M for **2**, 3.7 × 10^−6^ M for **3** and 3.4 × 10^−6^ M for **4**.

Dimeric species **2**–**4** have been investigated as suitable candidates as fluorescent membrane probes due to their distinguished solvatochromic behavior and good QY in apolar environment compared to **1** and **5**. Several experiments were carried out on **2**, **3** and **4** to verify this hypothesis, evaluating their emissive features upon the interaction with a lipidic model membrane. 1,2-dioleoyl-*sn*-glycero-3-phosphocholine (DOPC) and 1,2-dipalmitoyl-*sn*-glycero-3-phosphocholine (DPPC) large unilamellar vesicles (LUVs) were used to simulate two differently ordered lipid phases at 25 °C, a liquid disordered (L_d_) and a solid ordered (S_o_) one, respectively. Intercalation kinetic experiments were run, at first to evaluate the probes fluorescence upon interaction with the liposome bilayer membrane over time (Figure 4).

**Figure 5:**
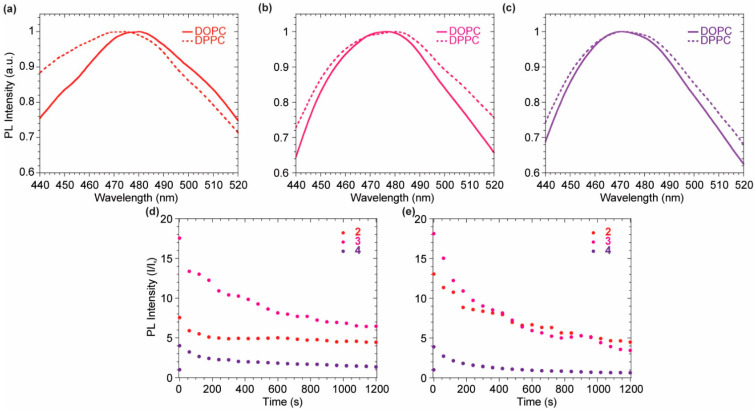
(**a**–**c**) Normalized emission profile in DOPC (solid line) and DPPC (dashed line) for compounds **2** (**a**), **3** (**b**), **4** (**c**). (**d**) Emission intensity vs. time in DOPC for **2** (red), **3** (magenta), **4** (purple). (**d**,**e**) Emission intensity vs. time DPPC for **2** (red), **3** (magenta), **4** (purple).

The quenched fluorescence signal in buffer media rapidly increases as soon as the probe intercalates in the lipidic bilayer, highlighting a successful and immediate response to the environmental change. Upon an initial remarkable increase of the emission signal due to the probe intercalation, the system equilibrates displaying a lower emission intensity for all probes (Figure 5d–e). This might be due to the lack of a polar or charged anchoring group on the probes able to stabilize the fluorophores inside the membrane by additional electrostatic or hydrogen bond interactions with the hydrophilic head of the outer layer. The interaction of each probe with the vesicles is almost immediate, allowing a strong fluorescence turn-on. The maximum emission wavelength is not affected by the lipid phase for probes **3** and **4**, while a bathochromic shift can be observed comparing the emission of probe **2** in DOPC with the one in DPPC (Figure 5a–c). The tightly packed organization of the lipid molecules in the DPPC S_o_ phase allows the probe to experience a more hydrophobic environment when compared to the interaction with DOPC LUVs. The L_d_ mobility of DOPC makes the probes surrounding more polar, bathochromically shifting the emission spectrum. Upon the initial incorporation in the bilayer membrane, driven by hydrophobic interaction, the decrease in the fluorescence signal, observed in the initial 20 min, suggested a slow partial release of the probes from the bilayer membrane. This might be explained considering a destabilization of the supramolecular lipidic environment by the deplanarized molecular conformation of the dimeric probes **2**–**4** (Figure 5d,e). Nevertheless, after 20 min, stable fluorescence signals were present for all the probes, indicating a positive staining of the membranes. It is worth noting that among the three investigated molecules, the asymmetric scaffold of **3** was the best performing since it had higher intensity, compared to the poorly soluble **2**, and higher signal to noise (I/I_0_) when compared to the brighter **4**.

**Figure 6:**
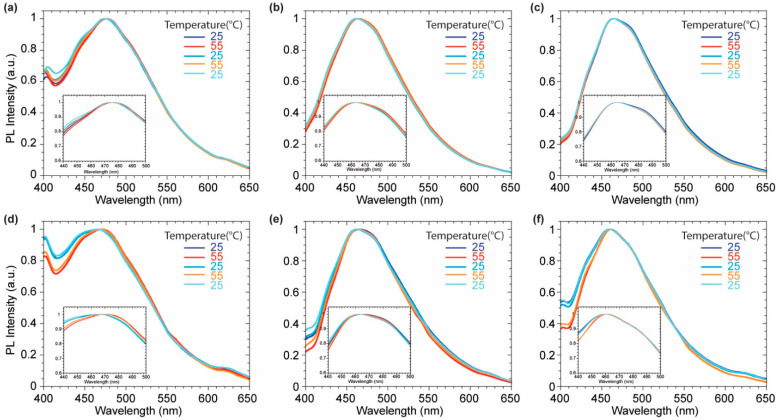
Normalized emission spectra of compounds **2** (**a**,**d**), **3** (**b**,**e**), **4** (**c**,**f**) in DOPC (**a**–**c**) and DPPC (**d**–**f**) over the heating cycles.

Upon the equilibration, the intercalated probes into the liposome membranes were subjected to several heating-cooling cycles, since DPPC undergo to a phase transition at 41 °C [39]. The probes were warmed up to 55 °C and then cooled down to 25 °C several times to assess their response to the change of membrane fluidity. DOPC liposomes were also tested as negative control, since they exhibit no transition phase in the explored temperature range. Probes **2** was showing the most promising results to monitor the membrane phases (Figure 6). The DPPC phase transition, driven by temperature, is characterized by higher system fluidity and higher hydration of the membrane which led to a more polar micro-environment reflected in a bathochromic shift of the emission signal. The complete reversibility of this phenomenon allows to exclude any chemical transformations driven by the higher thermal energy given to the probe. The emission profile of **2** was not affected by any heating-cooling cycle in DOPC since no phase transition occurs between 25 °C and 55 °C confirming the central role of the lipidic phase in the probe emissive response. The lack of changes in the emission profiles of probes **3** and **4** during the warm-cool cycles might be related to the presence of the pyridyl substituents on the imidazopyridine core. As known by literature [40], the pyridine group might reduce the degree of deplanarization via a hydrogen-bonds based pattern, while the presence of two pending phenyl rings might confer to **2** higher sensitivity to the variation lateral pressure inside the membrane which should drive the probe planarization.

**Figure 7:**
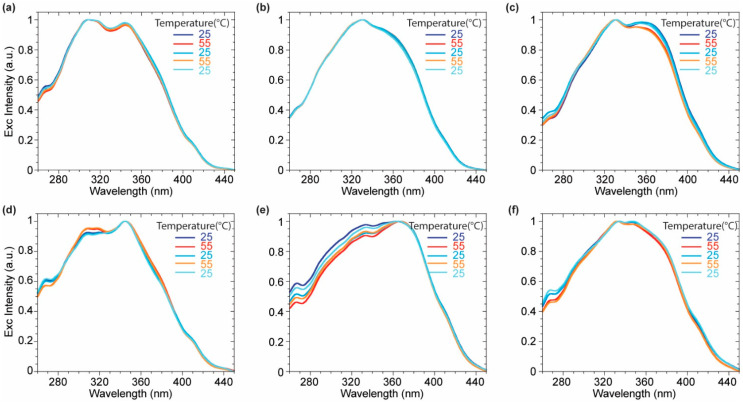
Normalized excitation spectra of compounds **2** (**a**,**d**), **3** (**b**,**e**), **4** (**c**,**f**) in DOPC (**a**–**c**), and DPPC (**d**–**f**) over the heating cycles.

Further support for this explanation can be found observing the normalized excitation spectra (Figure 7). While no significant differences occurred for probes **3** and **4**, **2** showed a variation in the relative intensities of two main contributions upon the temperature change. The blue-shifted band became more intense at 55° and decreased at 25 °C due to a greater contribution of the red-shifted planarized band in the S_o_ phase. The reduced Stokes shift of **2** in DPPC at low temperature, originating from a more conjugated ground-state and a blue-shifted emission might be explained considering the presence, at the same time, of the planarization effect induced by the membrane lateral pressure and less polar microenvironment arising from a lower hydration of the membrane bilayer. On the other hand, **2** did not highlight any variation either on the emission or on the excitation spectra recorded in DOPC where no significant variation of the fluidity or the hydration was expected. Additionally, the partition coefficient between the two explored lipid phases (DOPC and DPPC at rt) and water was evaluated for the asymmetrical probe **3**. The fluorescence enhancement by titration with increasing amounts of liposomes was monitored (Figures S3 and S4) and elaborated providing the partition coefficient value according to a procedure reported in literature for commercial fluorescent membrane probes [41]. In particular, the intensities of the emission spectra maxima were fitted versus the liposome’s concentration (Figure S4) according to Equation (1) reported in the material and method section. Finally, the partition coefficient K_R_ was obtained as the slope of the linear fit according to Equation (2) of the double reciprocal plot of the fluorescence intensity versus the liposome concentration (Figure S3 inset). The partition coefficient K_R_ in DOPC (9.6 × 10^5^) resulted to be slightly higher than the one in DPPC (6.0 × 10^5^). The tightly packed organization of phospholipids in DPPC makes less favorable the **3** partition in lipid phase compared to the liquid disordered DOPC phase. A comparison with partition coefficient of commercial probes (1,6–Diphenyl-1,3,5–hexatrien-DPH, 4-(4-didecylaminostyryl)-N-methylpyridiniuimodide–4-di-10-ASP) exhibiting K_R_ in DPPC around 1–3 × 10^6^ [41] values highlights the remarkable partitioning effect of the imidazo[1,5-*a*]pyridine in the lipidic bilayer, strongly encouraging further studies on these family of fluorophores.

## 3. Materials and Methods

### 3.1. General Methods

Reagents were purchased from Sigma-Aldrich and TCI Chemicals and were used without further purification. 1,2-dioleoyl-*sn*-glycero-3-phosphocholine (DOPC) and 1,2-dipalmitoyl-*sn*-glycero-3-phosphocholine (DPPC) were purchased from Avanti Polar Lipids. Solvents were purchased from VWR and Sigma-Aldrich and used as received. NMR solvents were purchased from Sigma-Aldrich and Euriso-top. H_2_O was purified with a Millipore RiOs 3 Water System. All reactions were monitored with analytical TLC (Merck Kieselgel 60 F254). Column chromatography was carried out with Biotage Isolera and Buchi Pure C-850 FlashPrep with silica gel particle size 60 μm. NMR spectra were obtained on a 600 MHz at Department of Drug Science and Technologies at the University of Turin and are reported as chemical shifts (δ) in ppm relative to TMS (δ = 0). Spin multiplicities are reported as a singlet (*s*), broad singlet (*bs*), doublet (*d*), doublet of triplets (*dt)*, triplet (*t*), triplet of doublets (*td*), quartet (*q*) and quintet (*quint*) with coupling constants (*J*) given in Hz, or multiplet (*m*). ESI-HRMS electrospray ionization high-resolution mass experiments were obtained with a Thermo Orbitrap Fusion mass spectrometer. Lipid Vesicles were prepared with a Mini-Extruder from Avanti Polar Lipids (pore size 100 nm). All the photophysical measurements were carried out in a 1 cm four-sided quartz cuvette from Hellma Analitics. Absorption spectra were measured on a Shimadzu UV-1900i UV-Vis Spectrophotometer, using a resolution of 0.5 nm. Steady state emission spectra were measured on a Shimadzu RF-6000. The excitation and the emission slits were set at 5 and 10 nm, respectively, while the resolution at 1 nm.

### 3.2. Synthetic Procedures

Products **1**, **2**, **4,** and **5** were prepared as previously reported (**1 [20]**, **2** [35], **4 [35]** and **5 [35]**).

*Bis-imidazo[1,5-a]pyridin-3-yl)benzene* (**3**). A mixture consisting of di(2-pyridyl)ketone (0.73 mmol, 1 eq.), terephthalaldehyde (1.46 mmol, 2 eq.), and ammonium acetate (21.85 mmol) in 25 mL of glacial acetic acid was stirred at 118 °C. After 6 h, the reaction mixture was added with (phenyl(pyridin-2-yl)methanone (0.73 mmol, 1 eq.). After 12 h, the reaction mixture was cooled to room temperature and the acetic acid was removed by evaporation under vacuum. The solid was dissolved in a saturated aqueous solution of Na_2_CO_3_ (40 mL) and the mixture was extracted with CH_2_Cl_2_ (3 × 30 mL). The organic layer was separated and the solvent evaporated under vacuum. The yellow formed solid was washed several times with diethyl ether (3 × 30 mL) and dried under vacuum. The obtained crude product was purified via column chromatography on silica gel (CH_2_Cl_2_-CH_3_OH 98:2) to get **3** as a yellow solid (0.51 g, 78%).

^1^H-NMR (600 MHz, CDCl_3_): δ = 8.78 (1H, d, ^3^J(H,H) = 9.2 Hz), 8.67 (1H, d, ^4^J(H,H) = 4.9 Hz), 8.34 (2H, dd, ^3^J(H,H) = 7.1 Hz, J(H,H) = 10.98 Hz), 8.30 (1H, d, ^3^J(H,H) = 6.9 Hz), 8.06 (m, 4H), 7.96 (2H, d, ^3^J(H,H) = 6.9 Hz), 7.89 (1H, d, ^3^J(H,H) = 9.3 Hz), 7.77 (1H, t, ^3^J(H,H) = 7.6 Hz), 7.49 (2H, t, ^3^J(H,H) = 7.5 Hz), 7.33 (1H, ^3^J(H,H) = 7.8 Hz), 7.15 (1H, dd, ^3^J(H,H) = 6.6 Hz, ^4^J(H,H) = 4.2 Hz), 7.00 (1H, dd, ^3^J(H,H) = 9.3 Hz, ^4^J(H,H) = 6.3 Hz), 6.85 (1H, dd, 3J(H,H) = 9.3 Hz, 4J(H,H) = 5.3 Hz), 6.74 (1H, t, 3J(H,H) = 6.7 Hz), 6.67 (1H, t, 3J(H,H) = 6.0 Hz) ppm; ^13^C NMR (150 MHz, CDCl_3_) δ 149.03, 149.02, 149.00, 137.51, 137.50, 136.47, 134.91, 132.59, 130.65, 130.56, 130.33, 128.87, 128.84, 128.80, 128.15, 126.96, 126.79, 122.06, 121.84, 121.71, 121.44, 120.69, 120.12, 120.06, 119.40, 114.43, 113.76. HRMS (ESI): *m*/*z* calculated for C_31_H_22_N_5_ [(M + H^+^)] 464.1877; found 464.1870. Decomposition at 300 °C before melting.

### 3.3. Optical Features

Absorption and fluorescence spectra. Stock solutions were prepared in DMSO with a concentration between 0.5 × 10^−3^ M and 2.3 × 10^−3^ M for probes, while probe 2 was dissolved in CHCl_3_ (0.7 × 10^−3^ M). The samples concentration was adjusted to have an absorbance between 0.1 and 1 at the $\lambda_{abs\;max}$ to evaluate the general photophysical properties in several organic solvents (molar extinction coefficient, $\lambda_{abs\;max}$ and $\lambda_{em\;max}$). All the absorption and steady state emission spectra were corrected for their respective blank.

Fluorescence quantum yield evaluation. The sample concentrations were adapted to have an absorbance lower than 0.1 at the excitation wavelength (λ_ex_) using the above-mentioned stock solutions. The fluorescence quantum yields (Φ) were evaluated compared on an external standard, quinine sulfate (Φ = 0.546 in H_2_SO_4_ 0.5 M, λ_ex_ 366 nm) by applying the following equation.

$$\Phi =\Phi_{STD}\frac{I}{I_{STD}}\frac{Abs_{STD}}{Abs}\frac{n^{2}}{n_{STD}^{2}}$$

where Φ*_STD_* is the fluorescence quantum yield of the standard, *I* and *I_STD_* are the integrated area of the emission band of the sample and the standard, respectively. *Abs* and *Abs_STD_* are the absorbance at the excitation wavelength for the sample and the standard, respectively. *n* and *n_STD_* are the solvent refractive indexes of the sample and the standard solutions, respectively.

### 3.4. Experiments with Liposomes

DOPC-LUVs preparation. A fine lipid film was prepared by a slow rotary evaporation (30 °C) of a DOPC (25.0 mg, 0.03 mmol) solution in MeOH/CHCl_3_ 1:1 (2.0 mL), followed by a final draining (5 h) in vacuo. The prepared film was hydrated with 1.0 mL buffer (10 mM phosphate, 100 mM NaCl, pH 7.4) for 30 min at rt, subjected to freeze-melt cycles (7×, liquid N_2_, 40 °C water bath) and extrusions (17×) through a polycarbonate membrane (pore size, 100 nm) at rt. Final conditions: ~32 mM DOPC; 10 mM phosphate, 100 mM NaCl, pH 7.4. The vesicles were used by seven days from the extrusion.

*DPPC–LUVs preparation*. A fine lipid film was prepared by a slow rotary evaporation (40 °C) of a DPPC (22.5 mg, 0.03 mmol) solution in MeOH/CHCl_3_ 1:1 (2.0 mL), followed by a final draining (5 h) in vacuo. The prepared film was hydrated with 1.0 mL buffer (10 mM phosphate, 100 mM NaCl, pH 7.4) for 30 min at 55 °C, subjected to freeze-melt cycles (7×, liquid N_2_, 55 °C water bath) and extrusions (21×) through a polycarbonate membrane (pore size, 100 nm) at 55° C. Final conditions: ~31 mM DPPC; 10 mM phosphate, 100 mM NaCl, pH 7.4. The vesicles were used by seven days from the extrusion.

*Kinetic experiments*. In a typical procedure, to a 2900 μL buffer (10 mM phosphate, 100 mM NaCl, pH 7.4 at rt) in a quartz cuvette, DOPC LUVs (100 μL, 1.1 mM DOPC final) or DPPC LUVs (100 μL, 1.02 mM DPPC final) all the tested probes (20 μL of the DMSO stock solution used for the photophysical measurements. A solution of **2** was appositely prepared in DMSO with a concentration of 0.11 × 10^−3^ M) were added in separated experiments. Each solution was mixed at rt and monitored acquiring the emission spectra every 1 min in the 20 min immediately after the sample preparation.

*Temperature-dependent experiments*. In a typical procedure, to a 2900 μL buffer (10 mM phosphate, 100 mM NaCl, pH 7.4 at rt) in a quartz cuvette, DOPC LUVs (100 μL, 1.1 mM DOPC final) or DPPC LUVs (100 μL, 1.02 mM DPPC final) and all the tested probes (20 μL of the DMSO stock solution). A solution of **2** was appositely prepared in DMSO with a concentration of 0.11 × 10^−3^ M) were added in separated experiments. The solution was mixed at rt, then the emission and excitation spectra were acquired after the respective equilibration time at rt calculated by the time dependence measurements. The solution was kept at 25 ± 1 °C for 15 min before the spectra acquisition, the cuvette was then warmed to 55 ± 1 °C using a hot plate and a sand bath, the solution was kept at this temperature for 15 min before the spectra acquisition. Then the temperature was lowered down to 25 ± 1 °C and the spectra were acquired after 15 min. The here described temperature cycle was repeated a second time.

*Partition coefficient experiments*. In a typical procedure, different DOPC or DPPC LUVs (32 mM and 31 mM, respectively) aliquots were added to a solution of **3** (3 μM, 2.92 mL) in buffer (10 mM phosphate, 100 mM NaCl, pH 7.4 at rt). The fluorescence spectra were recorded at equilibrium after addition of lipids (30 min for both vesicles). Then, the maximum intensity (*F*) was plotted against the lipid concentration (*L*) for each prepared solution, according to the Equation (1), reported in [41]:

(1) $$F=\frac{F_{0}L}{\frac{55.6}{K_{rip}}+L}\;$$

where *F*_0_ is the maximum fluorescence resulting from the total probe incorporation into membrane and *K_rip_* is the partition coefficient. The double reciprocal plot of the fluorescence (*F*) and the lipid concentration (*L*) should give a linear fit, according to the following Equation (2):

(2) $$\frac{1}{F}=\left(\frac{55.6}{K_{rip}F_{0}}\right)\frac{1}{L}+\frac{1}{F_{0}}\;$$

Finally, *K_rip_* can be calculated from the slope of the linear fit reported in Equation (2), using the *F*_0_ value calculated from the intercept of the fit.

## 4. Conclusions

In this work, we described the synthesis of a series of fluorescent probes based on the imidazo[1,5-*a*]pyridine core. Both monomeric **1** and **5** and extended structures **2**–**4** were prepared and an asymmetric version, **3**, was herein reported for the first time. The obtained probes were optically characterized in several organic solvents highlighting the wide Stokes shift and the peculiar features of the imidazo[1,5-*a*]pyridine based compounds. The presence of a pyridinil substituent in **2**–**4** enhances the quantum yield from 0.12 of **2** up to 0.38 of **4**, passing through 0.18 for **3** in apolar environment. The solvatochromic response of the most extended probes made them suitable candidate for the application as membrane bilayer probe. DOPC and DPPC LUVs were used to simulate the biological membrane: although all the tested compounds successfully interact with liposomes, probe **2** can discriminate between different lipid phases according to an emission profile shift. Nevertheless, the reported preliminary investigation in liposomes have suggested that the probe scaffolds require molecular modifications such as the introduction of polar or charge moieties to enhance the interaction and stabilization inside the membrane layers. Moreover, stronger push-pull character along the probe scaffold might be suitable to improve the membrane phases discrimination considering the different polarity and hydration degree among the membrane phases. This preliminary study encourages further detailed investigation of these class of compounds as fluorescent membrane probes, evaluating the electronic effects of functional groups on the core and exploring several heterogeneous liposomal model systems.

## Figures and Tables

**Table 1:** Solvatochromic properties of probes **1**–**5**.

Compound	Solvent	λ_abs_ [nm]	ε [M^−1^ cm^−1^]	λ_em_ [nm]	Stokes Shift [cm^−1^ (nm)]	QY ^a^
**1**	Dioxane	300	17,430	482	12,586 (182)	0.10
	DMF	288	16,280	480	13,889 (192)	0.13
	Ethyl Acetate	302	12,980	480	12,279 (178)	0.14
	Methanol	293	12,770	481	13,340 (188)	0.13
	THF	301	10,580	479	12,346 (178)	0.17
	Toluene	305	12,315	482	12,040 (177)	0.20
**2**	Dioxane	377	29,066	488	6033 (111)	0.11
	DMF	379	26,470	479	5508 (100)	0.08
	Ethyl Acetate	375	30,205	484	6006 (109)	0.08
	Methanol	370	23,960	474	5930 (104)	0.10
	THF	379	23,910	484	5724 (105)	0.11
	Toluene	382	23,810	486	5602 (104)	0.12
**3**	Dioxane	376	23,760	483	5892 (107)	0.13
	DMF	377	17,430	471	5294 (94)	0.12
	Ethyl Acetate	375	22,130	478	5746 (103)	0.11
	Methanol	368	16,305	464	5622 (96)	0.08
	THF	374	13,180	470	5461 (96)	0.09
	Toluene	378	16,820	480	5622 (102)	0.18
**4**	Dioxane	380	33,950	478	5395 (98)	0.29
	DMF	384	28,660	451	3869 (67)	0.17
	Ethyl Acetate	379	33,795	472	5199 (93)	0.24
	Methanol	369	27,475	460	5361 (93)	0.15
	THF	384	30,248	482	5295 (98)	0.26
	Toluene	383	28,922	470	4833 (87)	0.38
**5**	Dioxane	322	28,980	460	9317 (138)	0.13
	DMF	322	19,240	461	9364 (139)	0.18
	Ethyl Acetate	326	24,707	459	8888 (133)	0.16
	Methanol	313	18,270	457	10,067 (144)	0.16
	THF	319	18,138	460	9609 (141)	0.19
	Toluene	325	20,745	461	9077 (136)	0.17

All photophysical values reflect the average of three independent measurements. ^a^ Φ_F_ was measured referring to Quinine sulfate as standard (Φ_F_ = 0.546 in H_2_SO_4_ 0.5 M, λ_ex_ 366 nm).

## Data Availability

Data is contained within the article or Supplementary Materials.

## Supplementary Materials

The following supporting information can be downloaded at: https://www.mdpi.com/article/10.3390/molecules27123856/s1. Figure S1: E_T30_ plot; Figure S2: Absorption (dashed) and emission (solid) spectra of probes **2** (a), **3** (b) and **4** (c) in several mixtures THF/water. The emission spectra were normalized at 0.1 intensity at the excitation wavelength; Figure S3: Emission spectra of probe **3** at increasing concentrations of (a) DOPC and (b) DPPC. Inset: titration linearization plot; Figure S4: Emission maximum of probe **3** as a function of DOPC (green) and DPPC (blue) concentration. Figure S5: ^1^H-NMR of compound **3** in CDCl_3_; Figure S6: H,H COSY-NMR of **3** in CDCl_3_; Figure S7: ^13^C-NMR of compound **3** in CDCl_3_; Figure S8: HMRS spectrum of compound **3**; Figure S9: Excitation spectra of the compounds **2** (a,d), **3** (b,e), **4** (c,f) in DOPC (a–c) and DPPC (d–f) during the heating cycles; Figure S10: Emission spectra of the compounds **2** (a,d), **3** (b,e), **4** (c,f) in DOPC (a–c) and DPPC (d–f) during the heating cycles; Table S1: E_T30_ values and emission wavenumbers of the synthesized probes.

## Sample Availability

Samples of the compounds are available from the authors.
