# Supplementary material for: Imidazo[1,5-a]pyridine-Based Fluorescent Probes: A Photophysical Investigation in Liposome Models
Source: Molecules. 2022 Jun 16;27(12):3856. doi: 10.3390/molecules27123856 (PMC9230927; doi:10.3390/molecules27123856)
Supplement: Supplementary file 1 [file molecules-27-03856-s001.zip › molecules-1740958-supplementary.pdf]

Supplementary Information

Imidazo[1,5-a]pyridine-Based Fluorescent Probes: A Photophysical Investigation in Liposome Models

Giacomo Renno,[1,2] Francesca Cardano,[1] Giorgio Volpi,[1*] Claudia Barolo,[1,2] Guido Viscardi,[1] Andrea Fin[3*]

[1] Dipartimento di Chimica, Università degli Studi di Torino, via P. Giuria 7, 10125 Torino, Italy; giacomo.renno@unito.it, giorgio.volpi@unito.it, claudia.barolo@unito.it, guido.viscardi@unito.it

[2] NIS Interdepartmental Center, NIS Interdepartmental Centre and INSTM Reference Centre, Università degli Studi di Torino, Via Gioacchino Quarello 15/a, 10125 Torino, Italy

[3] Department of Drug Science and Technology, University of Turin, via P. Giuria 9, 10125 Torino, Italy; andrea.fin@unito.it

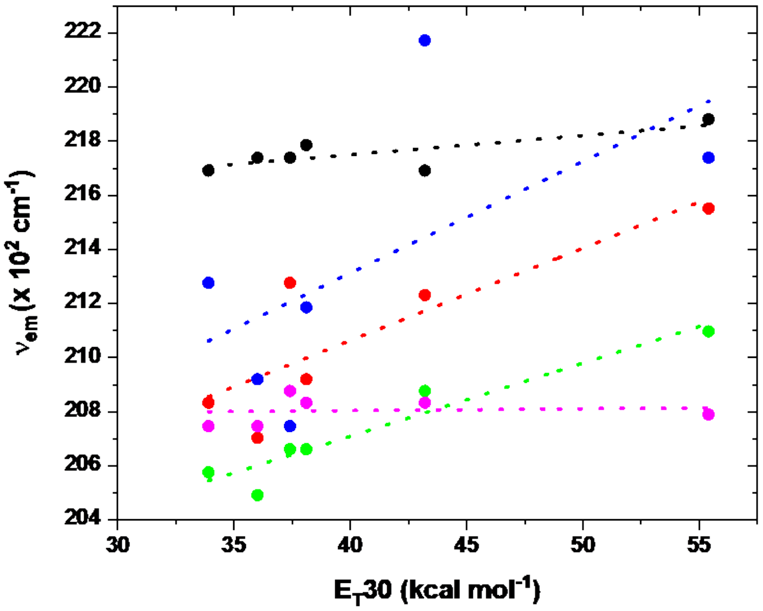

Figure S1. Emission wavenumber *vs* E_{T30} polarity scale for probe **1** (pink), **2** (green), **3** (red), **4** (blue), **5** (black). All values used for this plot are collected in Table S1.

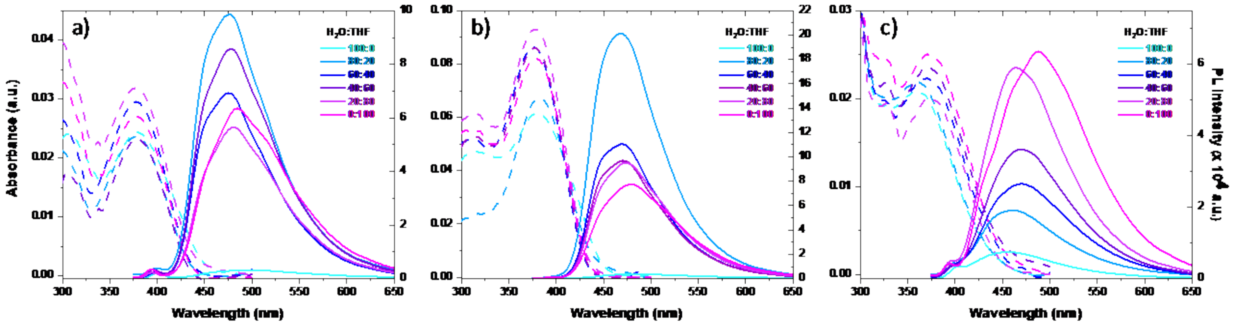

Figure S2. Absorption (dashed) and emission (solid) spectra of probes 2 (a), 3 (b) and 4 (c) in several mixtures THF/water. The emission spectra were normalized at 0.1 intensity at the excitation wavelength.

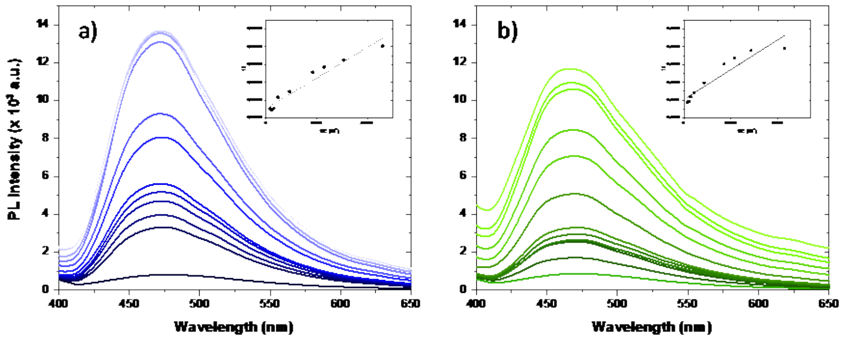

Figure S3. Emission spectra of probe **3** at increasing concentrations of (a) DOPC and (b) DPPC. Inset: titration linearization plot.

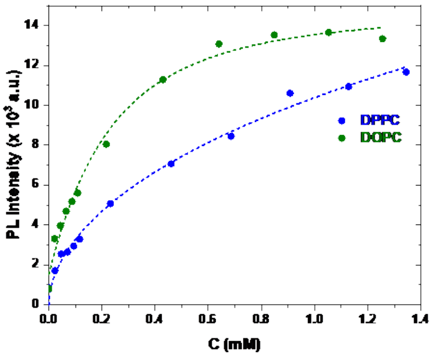

Figure S4. Emission maximum of probe **3** as a function of DOPC (green) and DPPC (blue) concentration.

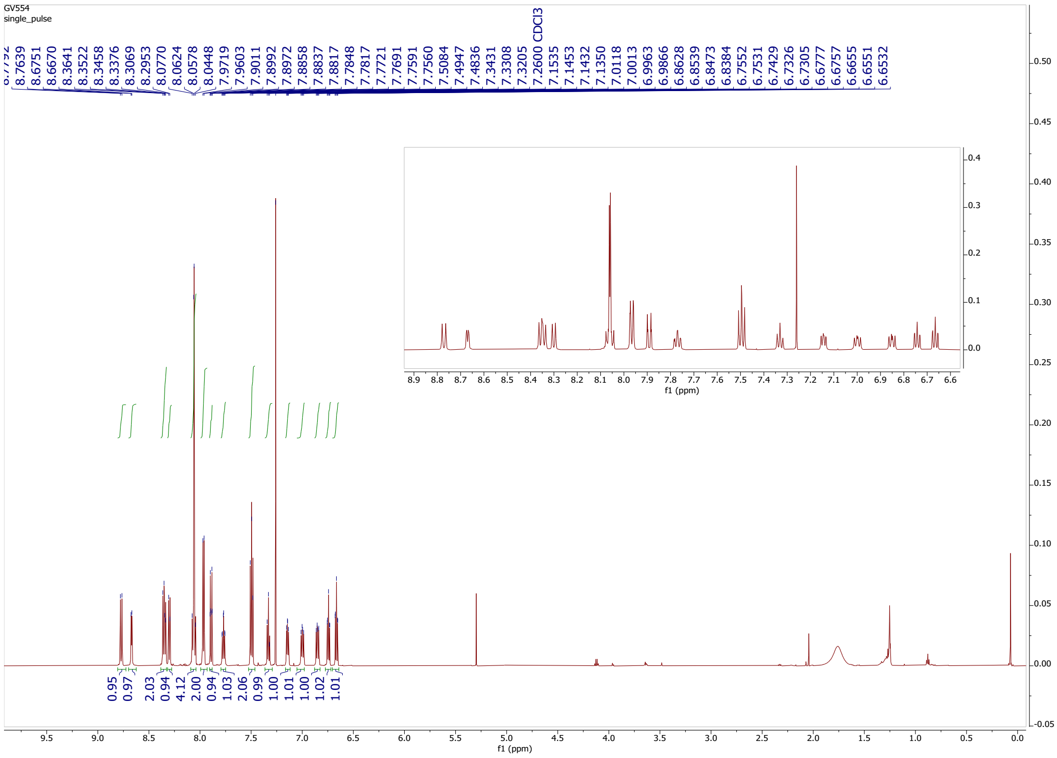

Figure S5. ^{1}H - NMR of compound **3** in $CDCl_3$.

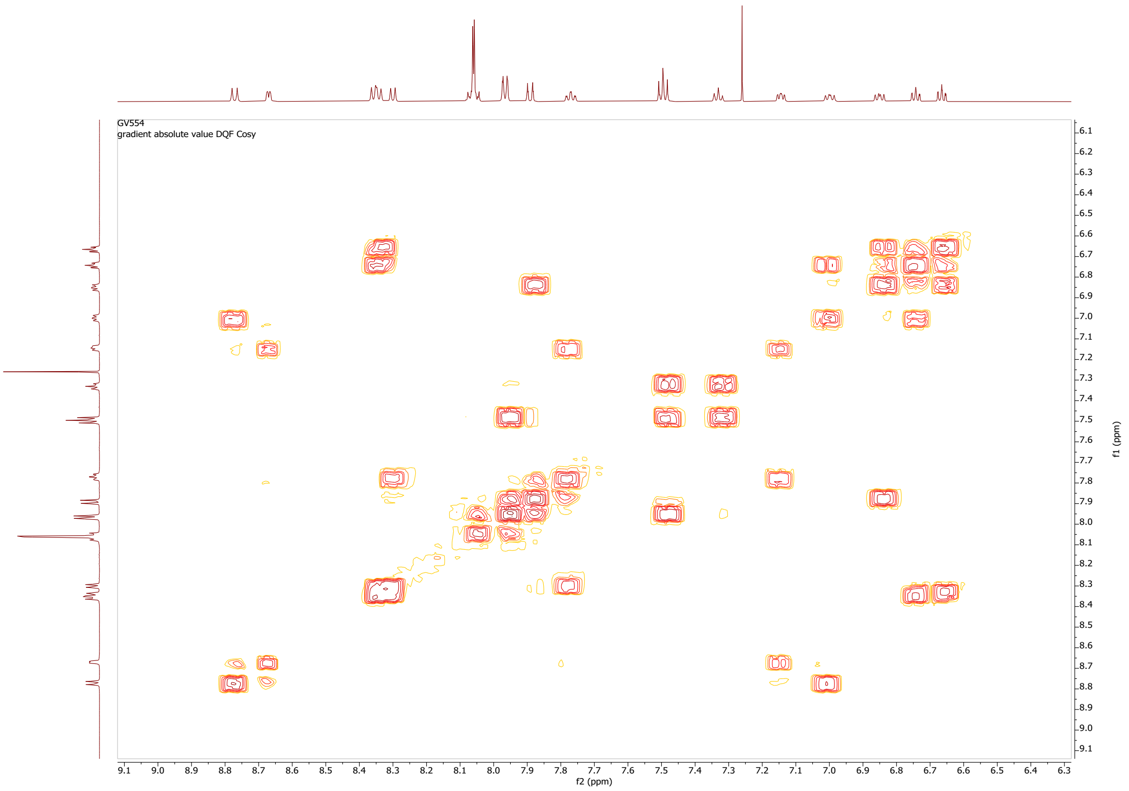

Figure S6. H,H - COSY - NMR of compound **3** in $CDCl_3$.

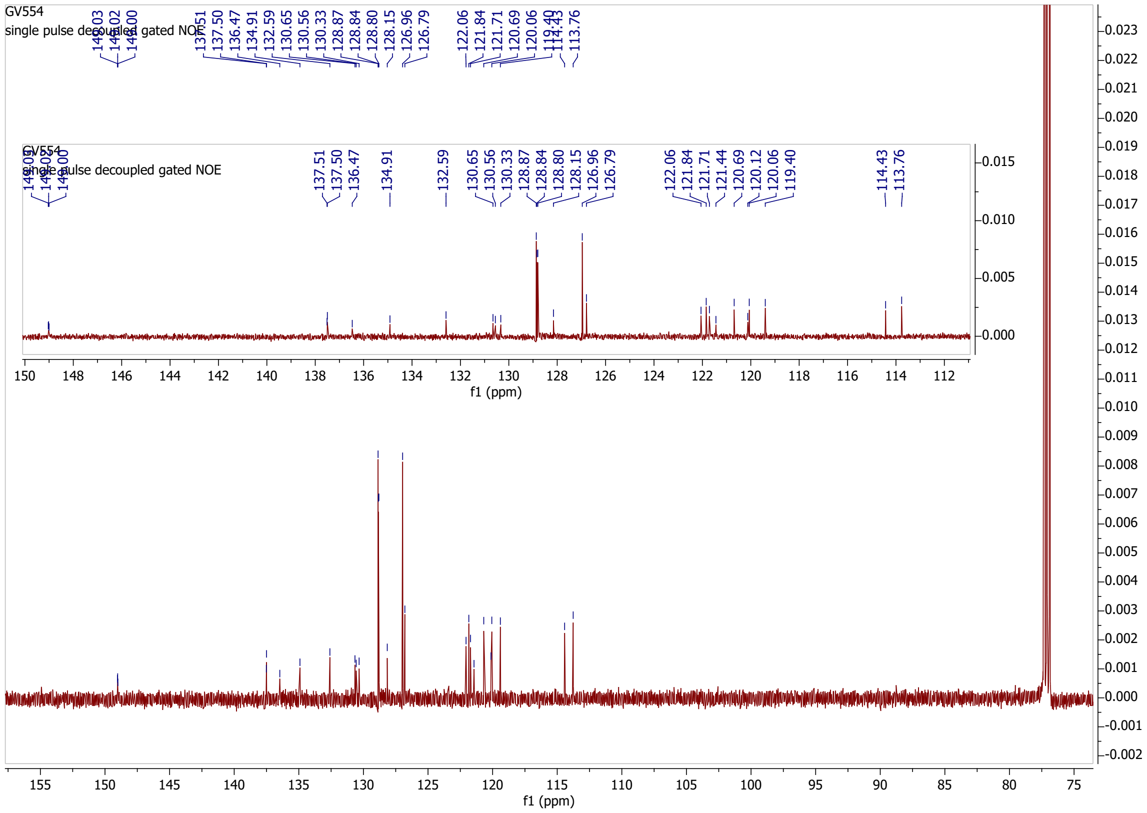

Figure S7. ^{13}C - NMR of compound **3** in $CDCl_3$.

Label	Formula	Computed Exact *m/z*	Accurate *m/z*	Delta ppm	RDB
**GV554**	$C_{31}H_{22}N_5$	464.1870	464.1877	1.460	23.5

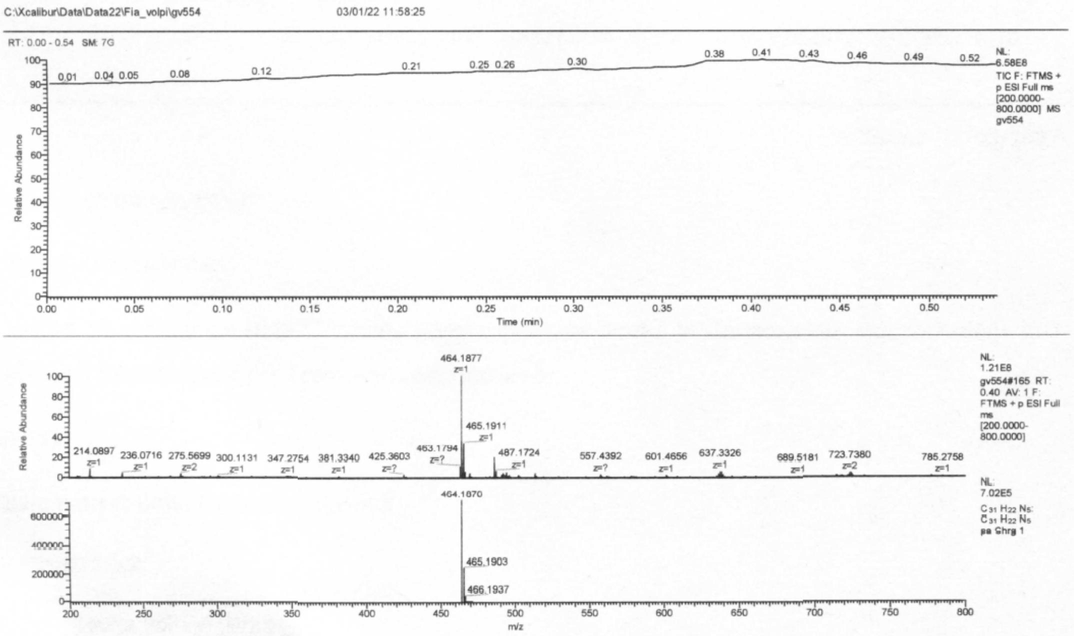

Figure S8. HRMS spectrum of probe **3.**

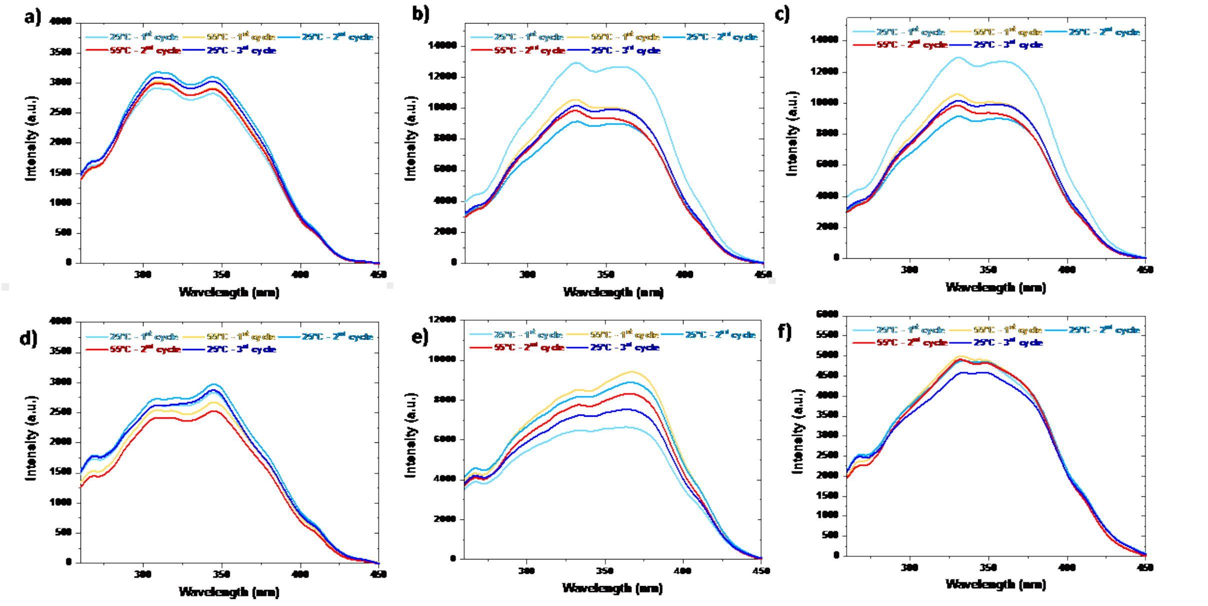

Figure S9. Excitation spectra of the compounds **2** (a,d), **3** (b,e), **4** (c,f) in DOPC (a-c) and DPPC (d-f) during the heating cycles.

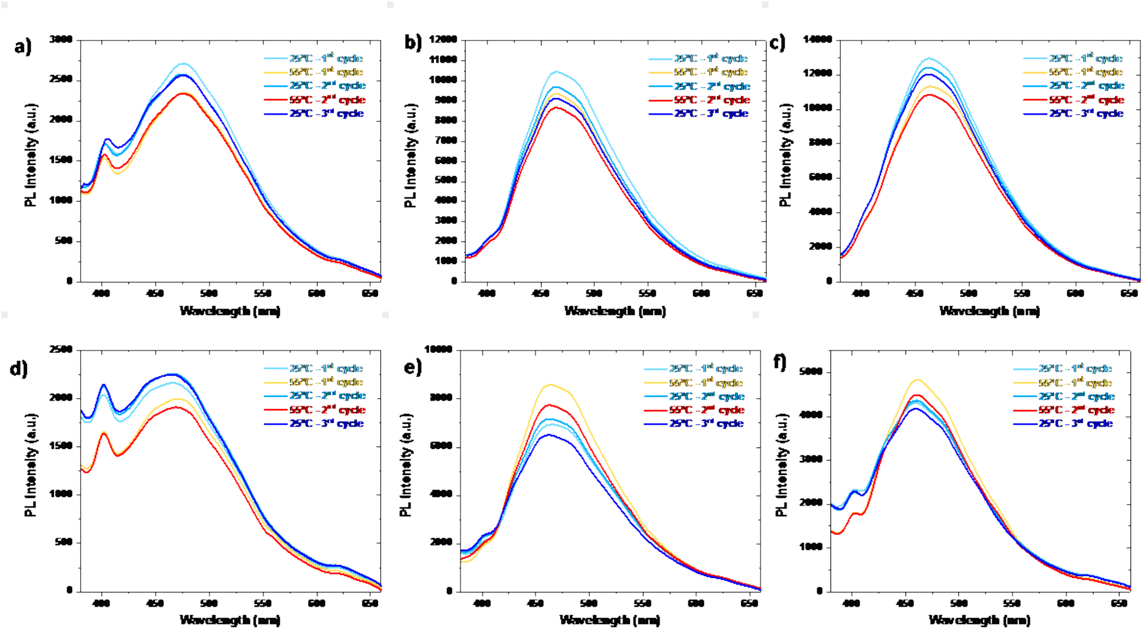

Figure S10. Emission spectra of the compounds **2** (a,d), **3** (b,e), **4** (c,f) in DOPC (a-c) and DPPC (d-f) during the heating cycles.

Table S1. E_{T30} values and emission wavenumbers of the synthesized probes.

		1	2	3	4	5
**Solvent**	***E_{T30} (kcal mol^{-1})*** [a]	**ν_{em} (cm^{-1})**	**ν_{em} (cm^{-1})**	**ν_{em} (cm^{-1})**	**ν_{em} (cm^{-1})**	**ν_{em} (cm^{-1})**
Dioxane	36.0	20747	20492	20704	20921	21739
Ethyl Acetate	38.1	20833	20661	20921	21186	21786
DMF	43.2	20833	20877	21231	22173	21692
THF	37.4	20877	20661	21277	20747	21739
Toluene	33.9	20747	20576	20833	21277	21692
Methanol	55.4	20790	21097	21552	21739	21882

[a] The parameters used are reported in ref. S1.

S1. Reichardt, C., Solvatochromic Dyes as Solvent Polarity Indicators, *Chem. Rev.*, **1994**, *94*, 2319-2358.
